# Predictive Value of Uric Acid/Albumin Ratio for Postoperative Atrial Fibrillation Following Isolated Coronary Artery Bypass Surgery

**DOI:** 10.21470/1678-9741-2024-0348

**Published:** 2026-02-13

**Authors:** Ilhan Koyuncu, Saadet Aydın, Ecem Gurses, Fatih Sivri, Zeynep Yapan Emren, Ilker Gul

**Affiliations:** 1 Cardiology Department, University of Bakircay Medical School, Izmir, Izmir, Turkey

**Keywords:** Coronary Artery Bypass Grafts, Postoperative Atrial Fibrillation, Uric Acid Albumin Ratio.

## Abstract

**Introduction:**

Postoperative atrial fibrillation (POAF) is associated with an increased risk of morbidity and mortality. This study aims to investigate the predictive value of uric acid/albumin ratio (UAR) for POAF following isolated coronary artery bypass grafting (CABG).

**Methods:**

This retrospective study screened patients who underwent isolated CABG between June 2017 and January 2024. POAF was diagnosed using standard clinical criteria. UAR was calculated by dividing the uric acid level by the albumin value.

**Results:**

This study included a total of 396 patients, who were categorized into two groups: POAF- and POAF+. Among them, 321 patients (mean age: 61.7 ± 10.8 years; 74.7% male) belonged to the POAF- group, while 75 patients (mean age: 71.3 ± 10.04 years; 68.1% male) were in the POAF+ group. While there were no significant differences observed between the groups in terms of sex, those in the POAF+ group were statistically older. The univariate and multivariate regression analyses revealed that age, C-reactive protein, hypertension, serum uric acid level, and UAR are independent risk factors for POAF. In the receiver operating characteristics analysis, the UAR (area under the curve [AUC]: 0.775) was found to be a better indicator compared to uric acid (AUC: 0.649) and serum albumin (AUC: 0.606), with a sensitivity of 70.5% and a specificity of 69.2%.

**Conclusion:**

UAR was shown to be an independent risk factor for POAF following isolated CABG.

## INTRODUCTION

**Table:** 

Abbreviations, Acronyms & Symbols				
ACE	= Angiotensin converting enzyme		EF	= Ejection fraction
AF	= Atrial fibrillation		FEV1	= Forced expiratory volume in one second
ARB	= Angiotensin receptor blockers		Hb	= Hemoglobin
ASA	= Acetyl salicylic acid		HL	= Hyperlipidemia
AUC	= Area under the curve		HT	= Hypertension
BNP	= Brain natriuretic peptide		IL	= Interleukin
BUN	= Blood urea nitrogen		K	= Potassium
CABG	= Coronary artery bypass grafting		LDH	= Lactate dehydrogenase
CC	= Cross-clamping		Na	= Sodium
CI	= Confidence interval		OR	= Odds ratio
COPD	= Chronic obstructive pulmonary disease		POAF	= Postoperative atrial fibrillation
CPB	= Cardiopulmonary bypass		RCA	= Right coronary artery
Cr	= Creatinine		ROC	= Receiver operating characteristics
CRF	= Chronic renal failure		UAR	= Uric acid/albumin ratio
CRP	= C-reactive protein		WBC	= White blood cells
DM	= Diabetes mellitus			

Atrial fibrillation (AF) is the most frequently observed cardiac complication following coronary artery bypass grafting (CABG), with a prevalence of 20 - 40%. It can be particularly observed in 30 - 50% of cases involving valvular operation. It is often detected within the first three days post-surgery^[1]^. Postoperative atrial fibrillation (POAF) is typically linked to increased morbidity, mortality, heart failure, renal failure, extended hospitalization, and risk of thromboembolism^[2]^. Though the etiopathogenesis of POAF is not fully understood, older age, higher-than-normal brain natriuretic peptide levels, male sex, pre-existing comorbidities, prevalent heart failure, and chronic obstructive pulmonary disease (COPD) are recognized as risk factors^[3]^.

It has been demonstrated that systemic inflammation, oxidative stress, and increased neurohumoral activation play a key role in the development of POAF following cardiac surgery. Studies have shown that various pro-inflammatory cytokines, such as increased interleukin (IL)-6, IL-8, and tumor necrosis factor-alpha, along with inflammatory markers like C-reactive protein (CRP) and the platelet-lymphocyte ratio, have a prognostic role in POAF^[3]^.

Uric acid is the end product of purine nucleotide metabolism. Increased levels of serum uric acid have pro-oxidant and pro-inflammatory effects. Numerous studies have demonstrated a close relationship between hyperuricemia and cardiovascular diseases^[4]^. Albumin, on the other hand, is a negative acute-phase marker, whose synthesis decreases and breakdown increases in inflammatory conditions. The prognostic value of low albumin levels has been highlighted in several studies for a range of diseases such as coronary artery disease, cancer, and sepsis^[5]^. The investigation of the prognostic role of the increased uric acid/albumin ratio (UAR), resulting from elevated uric acid and decreased albumin, has recently emerged. Its prognostic value has been emphasized for many conditions, such as coronary artery disease, contrast nephropathy, and arrhythmia recurrence^[6]^. UAR has been identified as an independent predictor of new-onset AF in patients with ST-elevation myocardial infarction^[7]^. Furthermore, recent findings have demonstrated that elevated UAR levels are significantly associated with AF recurrence following cryoballoon catheter ablation^[8]^, exhibiting superior predictive value compared to other inflammatory markers^[9]^. However, no study in the literature has investigated the relationship between POAF and UAR.

The objective of this study is to examine the relationship between POAF and UAR.

## METHODS

After obtaining approval for the study protocol from the institutional ethics committee (Bakırçay University Non-Interventional Clinical Research Ethics Committee permission, number 1780), 750 patients who underwent isolated CABG (patients undergoing cardiopulmonary bypass) at a single center between 2017 and 2024 were retrospectively screened. The study was conducted in compliance with the Declaration of Helsinki.

**Fig. 1:**
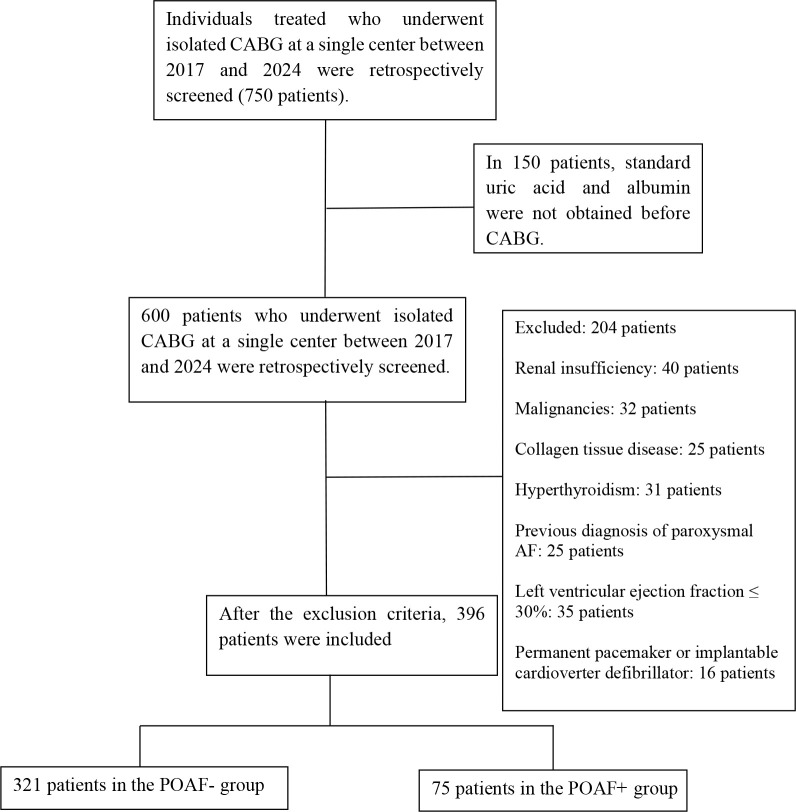
Selection of the study group is summarized in the study flow chart. AF=atrial fibrillation; CABG=coronary artery bypass grafting; POAF=postoperative atrial fibrillation.

The inclusion criteria for the study consisted of patients who underwent isolated CABG and aged older than 18 years. The exclusion criteria included patients with a history of unstable angina or myocardial infarction for < 7 days, inflammatory bowel disease, malignancy, arthritis, infections, hyperthyroidism, non-alcoholic fatty liver disease, preoperative AF, previous diagnosis of paroxysmal AF, the use of synthetic hormone preparations, steroids, thiazolidinediones, or propylthiouracil, the presence of a permanent pacemaker or implantable cardioverter defibrillator, the use of amiodarone and digitalis before a CABG surgery, hemodynamic instability prior to a CABG surgery, diagnosis of decompensated congestive heart failure, the need for urgent surgery, and the presence of chronic renal failure (CRF) with creatinine levels > 2 mg/dL. Moreover, patients undergoing valvular surgery or a second bypass surgery, as well as those with a left ventricular ejection fraction ≤ 30% were excluded from the study. Patients who received preoperative and postoperative albumin infusion were not included in the study. Following the exclusion criteria, a total of 396 patients were included in the current study. The selection of the study group is summarized in the study flow chart (Figure 1).

All patients were thoroughly questioned regarding hyperlipidemia (HL), diabetes mellitus (DM), tobacco use, asthma, and COPD. Detailed information on all medical treatments received by the patients was also collected. Hematological, biochemical, and serological values were obtained and recorded from peripheral blood samples after a 12-hour fasting period. A glomerular filtration rate < 60 mL/min for more than three months was considered CRF. As for DM, the criteria included the use of antidiabetic medications, at least two fasting blood glucose measurements > 126 mg/dL, or HbA1c levels > 6.5%. HL was confirmed in patients if low-density lipoprotein levels were > 160 mg/DL or if they were on statin therapy. The criteria for diagnosing COPD included an forced expiratory volume in one second (FEV1)/forced vital capacity ratio < 70% post-inhalation of a bronchodilator or an FEV1 < 70%. Preoperative treatments for all patients were resumed on the first postoperative day.

Echocardiographic evaluation of all patients was performed using the iE33 cardiac ultrasound system (Philips Healthcare, Best, The Netherlands) and 2.5 - 5 MHz probes, and ejection fraction was measured using the modified Simpson method.

### Laboratory Analysis

The basal hematological and biochemical values of all patients included in the study were investigated and recorded via the electronic system.

### Diagnosis and Treatment of Postoperative Atrial Fibrillation

After the surgery was performed, all patients were continuously monitored for 72 - 96 hours using the Apexpro 7-lead system (General Electric Medical Systems). New-onset AF was defined as an irregular pulse lasting > 5 minutes. AF was diagnosed by the absence of P waves on a 12-lead electrocardiogram in patients under intensive care or service monitoring if they experienced palpitations or if an irregular pulse was detected during physical examination. In accordance with the prior treatment protocols, POAF was treated with intravenous amiodarone or oral beta-blockers. Anticoagulant therapy was also administered. In cases of hemodynamic instability, electrical cardioversion was performed.

### Statistical Analysis

The data obtained were analyzed using the IBM SPSS Statistics for Windows, version 21.0 (IBM Corp., Armonk, N.Y., USA) statistical software. The conformity of the continuous variables to a normal distribution was assessed using both visual - histogram and probability graphs - and analytical methods - Kolmogorov-Smirnov/Shapiro-Wilk tests. As for the descriptive statistics, the data following a normal distribution were presented as mean and standard deviation, while the non-normally distributed data were presented as median, minimum, and maximum values. A chi-square test was used in order to determine if there were differences between the categorical variables. To compare continuous variables within independent groups, Student’s *t*-test was applied for those with parametric properties, while the Mann-Whitney U test was used for non-parametric variables. In the univariate analysis, variables with a *P*-value < 0.20 and those considered clinically significant were included in the multivariate logistic regression model to adjust for potential confounding effects. A *P*-value < 0.05 was considered statistically significant. Lastly, receiver operating characteristics (ROC) curve analysis was used to determine the specificity and sensitivity of the UAR.

## RESULTS

This study includes a total of 396 patients, of which 321 (mean age: 61.7 ± 10.8 years; 74.7% male) were classified as the POAF- group, and 75 (mean age: 71.3 ± 10.04 years; 68.1% male) were classified as the POAF+ group. While no differences in sex were observed between the groups, the POAF+ group was statistically older. When the patients’ medical histories and treatments were compared, statistically higher rates of hypertension were observed in the POAF+ group. The baseline characteristics of the participants are presented in Table 1.

**Table 1:** Baseline clinical characteristics of patients.

	POAF-	POAF+	*P*-value
	(n = 321)	(n = 75)	
	Number of patients (%)	Number of patients (%)	
Demographic characteristics			
Age (years)	61.7 ± 10.8	71.3 ± 10.04	0.001
Male sex	240 (74.7)	51 (68.1%)	0.254
Smoking	130 (40.9)	31 (41.3)	0.254
Medical history			
DM	136 (42.3)	33 (44)	0.253
HT	252 (78.5)	66 (88)	0.001
HL	102 (31.7)	25 (33.3)	0.186
Stroke	14 (4)	5 (6.6)	0.321
COPD	65 (20.2)	16 (21.3)	0.214
Medical treatment			
ACE inhibitors	157 (49.5%)	36 (48.6%)	0.535
ARB	112 (35.0%)	28 (38.7%)	0.151
Beta blocker	67 (21.3%)	16 (22.5%)	0.892
Calcium channel blocker	77 (24.7%)	21 (28.2%)	0.652
Diuretics	83 (26.4%)	21 (29.0%)	0.146
Statins	58 (30.1)	24 (33.1)	0.258
ASA	75 (23.3)	18 (25%)	0.598

ACE=angiotensin converting enzyme; ARB=angiotensin receptor blockers; ASA=acetyl salicylic acid; COPD=chronic obstructive pulmonary disease; DM=diabetes mellitus; HL=hyperlipidemia; HT=hypertension; POAF=postoperative atrial fibrillation

When the laboratory and echocardiographic levels of the patients were examined, no differences between the groups were observed in terms of hemoglobin, white blood cells, and renal function levels. The POAF+ group had statistically higher levels of uric acid (7.8 ± 1.9 vs. 5.8 ± 1.6, P = 0.001), UAR (0.279 ± 0.31 *vs.* 0.141 ± 0.21, *P* = 0.001), and CRP (9.95 ± 5.54 *vs.* 7.21 ± 4.46, *P* = 0.001). In the POAF- group, however, albumin (3.1 ± 1.3 *vs.* 2.8 ± 1.8, *P* = 0.001) was found to be statistically higher. There were no notable variations in echocardiographic measurements between the groups (Table 2).

**Table 2:** Comparison of laboratory and echocardiographic values of the groups.

	POAF-	POAF+	*P*-value
	(n = 321)	(n = 75)	
Glucose (mg/dl)	187.2 ± 97.5	175.7 ± 62.8	0.512
WBC (uL)	11.8 ± 2.12	12.02 ± 1.3	0.125
Hb (mg/dl)	13.5 ± 1.4	12.5 ± 1.3	0.236
BUN (mg/dL)	31.2 ± 18.2	33.5 ± 19.9	0.214
Cr (mg/dL)	0.98 ± 0.15	0.72 ± 0.22	0.211
Na (mmol/L)	136.1 ± 2.7	139.2 ± 2.7	0.113
K (mmol/L)	4.34 ± 1.31	4.51 ± 1.23	0.156
Uric acid (mg/dL)	5.8 ± 1.6	7.8 ± 1.9	0.001
Albumin (g/l)	3.1 ± 1.3	2.8 ± 1.8	0.001
UAR	0.141 ± 0.21	0.279 ± 0.31	0.001
LDH (g/dL)	275.2 ± 154.1	287.25 ± 170.2	0.574
CRP (mg/dL)	7.21 ± 4.46	9.95 ± 5.54	0.001
Troponin (T) (ng/dl)	61.14 ± 29.11	58.7 ± 25.1	0.125
D-dimer (µg/mL)	2.72 ± 1.91	2.42 ± 1.2	0.122
EF (%)	58 ± 4.2	54 ± 6.9	0.654
Left atrium (mm)	35.93 ± 3.30	36.68 ± 3.47	0.324
Left atrial volume (ml)	56.5 ± 22.3	68.9 ± 19.8	0.587

Troponin T reference values (0-100)

BUN=blood urea nitrogen; Cr=creatinine; CRP=C-reactive protein; EF=ejection fraction; Hb=hemoglobin; K=potassium; LDH=lactate dehydrogenase; Na=sodium; POAF=postoperative atrial fibrillation; UAR=uric acid/albumin ratio; WBC=white blood cells

When intraoperative values were compared, no differences were detected between the groups (Table 3).

**Table 3:** Intraoperative and postoperative data.

	POAF-		POAF+	*P*-value
	(n = 321)		(n = 75)	
	Number of patients (%)		Number of patients (%)	
Number of distal anastomoses (n)	3.03 ± 0.88		3.10 ± 0.87	0.841
RCA bypass (%, n)	60 (18)		60 (6)	1.001
CPB time (min)	84.8 ± 17.8		93.4 ± 22.9	0.254
CC time (min)	58.2 ± 19.9	76.4 ± 34.3		0.456
Drainage (ml)	411.7 ± 199.4	440.0 ± 185.3		0.231
Extubation time (h)	6.83 ± 2.65	7.50 ± 3.60		0.711

CC=cross-clamping; CPB=cardiopulmonary bypass; POAF=postoperative atrial fibrillation; RCA=right coronary artery

In the univariate and multivariate regression analyses, age (odds ratio [OR]: 1.314, 95% confidence interval [CI]: 1.141 - 1.601, *P* = 0.001), hypertension (OR: 1.221, 95% CI: 1.121 - 1.324, *P* = 0.001), CRP (OR: 1.232, 95% CI: 1.101 - 1.338, *P* = 0.001), and UAR (OR: 2.704, 95% CI: 1.701 - 3.440, *P* = 0.001) were identified as independent risk factors for POAF (Table 4).

**Table 4:** Effects of different variables on POAF in univariate and multivariate logistic regression analysis.

	Univariate				Multivariate			
	OR	95% CI		*P*-value	OR	95% CI		*P*-value
		Lower	Upper			Lower	Upper	
Age	1.522	1.241	1.854	0.000	1.314	1.141	1.601	0.001
HT	1.212	1.102	1.410	0.000	1.221	1.121	1.324	0.001
HL	1.211	0.875	1.487	0.254				
ARB	1.004	0.989	1.009	0.541				
Diuretic	1.024	0.947	1.098	0.124				
CRP	1.122	1.012	1.312	0.000	1.232	1.101	1.338	0.001
Uric acid	1.112	0.951	1.225	0.001				
UAR	2.312	1.543	3.402	0.000	2.704	1.701	3.440	0.001
WBC	1.014	0.983	1.037	0.385				
Na	1.254	0.874	1.512	0.124				
K	1.124	0.921	1.325	0.698				
D-dimer	1.411	0.974	1.910	0.584				
Troponin	1.321	0.954	1.798	0.874				

ARB=angiotensin receptor blockers; CI=confidence interval; CRP=C-reactive protein; HL=hyperlipidemia; HT=hypertension; K=potassium; Na=sodium; OR=odds ratio; UAR=uric acid/albumin ratio; WBC=white blood cells

**Fig. 2:**
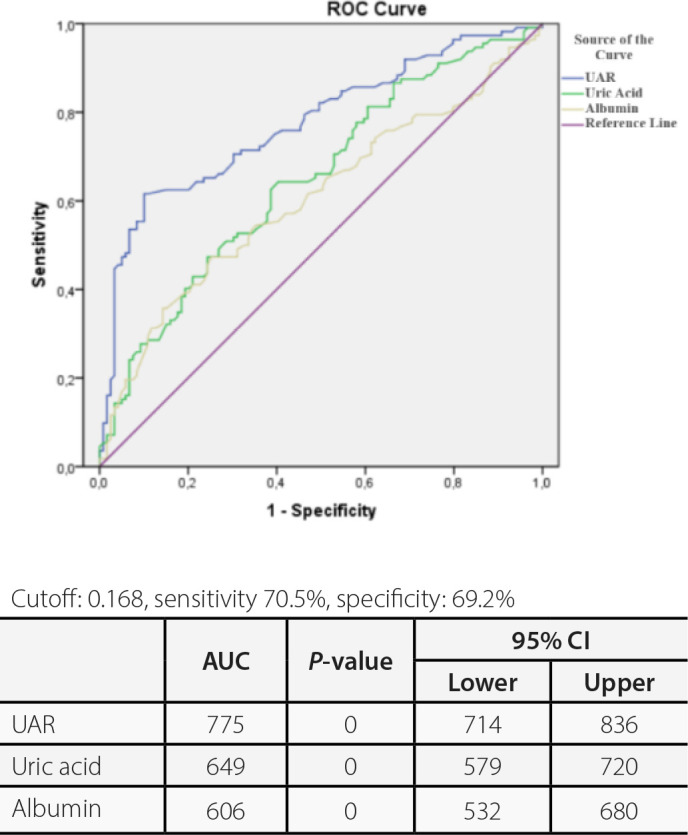
Uric acid/albumin ratio (UAR) receiver operating characteristics (ROC) analysis values. AUC=area under the curve; CI=confidence interval.

The ROC analysis revealed an area under the curve (AUC) of 0.775 for the uric acid ratio. It was determined that the UAR is a better indicator compared to uric acid (AUC: 0.649) and albumin (AUC: 0.606). We calculated that a cutoff point of 0.168 for UAR could estimate the presence of POAF with a sensitivity of 70.5% and specificity of 69.2% (Figure 2).

## DISCUSSION

This study is the first to investigate the predictive value of UAR on POAF. Our findings indicate that uric acid serves as an independent risk factor for the development of POAF following CABG, and UAR value > 0.168 was found to be a predictor of POAF.

Previous studies have similarly identified age, hypertension, CRP, and uric acid as independent risk factors for the development of POAF following CABG. The loss of myocardial fibers, atrial fibrosis, inflammation, and oxidative stress associated with aging constitute the fundamental pathophysiology of POAF following cardiac surgery^[2]^. In a single-center retrospective study involving 14.960 patients, a close relationship between aging and POAF was identified, with a five-fold increase observed in individuals over the age of 72 years compared to those aged 55 years^[10]^. Increased left atrial diameter, reduced atrial contraction, heightened oxidative stress, and inflammation due to uncontrolled hypertension constitute the fundamental pathophysiology of POAF following CABG. In their study, Mangi et al.^[11]^ found that hypertension was the most commonly observed comorbidity in patients experiencing POAF following CABG, with a prevalence of 68%. Numerous previous studies have shown that increased inflammation and oxidative stress are significant risk factors for POAF. In this regard, CRP is among the most important biomarkers^[12,13]^. In a study involving 6,711 patients conducted by Olesen et al.^[14]^, it was found that patients with higher-than-normal CRP levels had a risk 1.31 times greater than those with lower levels.

Increased inflammation and oxidative stress during bypass surgery are the most common causes of postoperative complications, especially POAF^[15]^. Numerous studies have shown the relationship between inflammation and POAF^[16]^. In very important pathophysiological studies, atrial biopsy results have shown increased inflammation, myocyte necrosis, and fibrosis in POAF patients^[17]^. Serum albumin is a negative acute phase reactant, and a decrease in its level is associated with adverse cardiac events^[18]^. Albumin is not only related to inflammation but also to increased blood viscosity and endothelial dysfunction. Numerous studies have shown the relationship between low serum albumin and POAF^[19]^. Research has highlighted the significance of hyperuricemia in relation to inflammation and oxidative stress. Increased inflammation and oxidative stress result in cardiac structural and electrophysiological changes^[20,21]^. Additionally, animal studies have shown decreased cardiomyocyte activity and increased remodeling in rats with elevated uric acid levels. Electrophysiological changes have been observed to cause atrial conduction disturbances and re-entry^[22]^. In another study by Zhang et al.^[23]^, it was observed that patients with high serum uric acid levels had a 1.493-fold greater risk of developing POAF following CABG than those with lower levels. The prognostic value of the UAR is a newly identified inflammation marker, whose predictive value has been demonstrated for various inflammatory diseases, including cardiovascular conditions^[24]^. In a study by Şaylık et al.^[25]^, UAR was shown to be an independent predictor of carotid intima-media thickness in individuals with existing hypertension. Similarly, Biter et al.^[26]^ demonstrated that higher-than-normal UAR is an independent risk factor for major cardiac and cerebral events in patients with aortic stenosis undergoing transcatheter aortic valve implantation (OR: 2.47). In their study on patients with ST-elevation myocardial infarction, Kalkan S. et al.^[27]^ also identified UAR as an independent risk factor (OR: 1.33). In another study by Oflar et al.^[28]^, UAR was shown to be an independent risk factor for the severity of peripheral artery disease. Similarly, Özgür et al.^[29]^ highlighted that high UAR is an independent risk factor for early mortality in patients with acute renal failure. Çakmak et al.^[30]^, on the other hand, found that, in patients with non-ST-elevation myocardial infarction, UAR might serve as an independent predictor for the spread of coronary artery disease. In a retrospective study by Li et al.^[31]^, which included 2,298 patients with a two-year follow-up, high UAR was identified as an independent risk factor for mortality in patients with unstable angina pectoris.

Inflammation-based biomarkers have attracted increasing interest in predicting POAF. As reported by Tekkeşin et al.^[32]^, the monocyte-to-high-density lipoprotein ratio was found to be significantly elevated in patients who developed POAF following aortocoronary bypass graft surgery, underscoring its role as a surrogate marker for systemic inflammation and oxidative stress. Similarly, the Morphology-Voltage-P-wave electrocardiogram risk score - which incorporates P wave morphology, voltage, and duration - has proven useful in identifying patients at risk for POAF, particularly in relation to different internal thoracic artery grafting strategies^[33]^. More recent data suggest that the UAR, due to its ability to reflect both pro-inflammatory burden and antioxidant reserve, may represent a superior predictive marker. This dual-pathway reflection positions UAR as a promising and more comprehensive biomarker for AF risk stratification^[8]^. Our findings also support a strong association between UAR and POAF, consistent with the current literature.

In our study, although well-established risk factors such as age, hypertension, and CRP were confirmed to be predictive of POAF, multivariate analyses revealed that UAR remained significantly associated with POAF even after adjusting for all other potential confounders. Furthermore, ROC curve analysis demonstrated that UAR had greater predictive value for POAF than either uric acid or albumin alone.

### Limitations

A key limitation of this study is that it was conducted at a single center with a small number of patients. Additionally, the patients’ surgery duration, inotropic support, and blood transfusion needs were not included in the study. Lastly, patients did not receive long-term follow-up after discharge from the hospital.

## CONCLUSION

With this study, it has been demonstrated for the first time that the UAR is an independent risk factor for POAF following CABG. Our findings suggest that high serum UAR should not be overlooked in identifying high-risk patients for POAF following CABG. We are of the opinion that more comprehensive and prospective studies could help us understand the relationship between UAR and POAF, as well as determine their diagnostic and therapeutic implications.

## Data Availability

The authors do not consent to publicly sharing the study data. Therefore, data sharing is not required.

## Artificial Intelligence Usage

The authors declare that no artificial intelligence tool was used in the preparation of this article.
